# Interferon sensitivity-determining region of hepatitis C virus influences virus production and interferon signaling

**DOI:** 10.18632/oncotarget.23562

**Published:** 2017-12-21

**Authors:** Ryuichi Sugiyama, Asako Murayama, Sayuri Nitta, Norie Yamada, Megumi Tasaka-Fujita, Takahiro Masaki, Hussein Hassan Aly, Masaaki Shiina, Akihide Ryo, Koji Ishii, Takaji Wakita, Takanobu Kato

**Affiliations:** ^1^ Department of Virology II, National Institute of Infectious Diseases, Tokyo, Japan; ^2^ Department of Gastroenterology and Hepatology, Tokyo Medical and Dental University, Tokyo, Japan; ^3^ Faculty of Medicine, Tokyo Medical and Dental University, Tokyo, Japan; ^4^ Department of Gastroenterology and Hepatology, Shin-Yurigaoka General Hospital, Kawasaki, Japan; ^5^ Department of Microbiology, Yokohama City University School of Medicine, Yokohama, Japan; ^6^ Present address: Department of Laboratory Medicine, The Jikei University School of Medicine, Nishi-shinbashi, Minato-ku, Tokyo, Japan

**Keywords:** HCV, IFN, innate immunity, cell culture, drug resistance, Immunology

## Abstract

The number of amino acid substitutions in the interferon (IFN) sensitivity-determining region (ISDR) of hepatitis C virus (HCV) NS5A is a strong predictor for the outcome of IFN-based treatment. To assess the involvement of ISDR in the HCV life cycle and to clarify the molecular mechanisms influencing IFN susceptibility, we used recombinant JFH-1 viruses with NS5A of the genotype 1b Con1 strain (JFH1/5ACon1) and with NS5A ISDR containing 7 amino acid substitutions (JFH1/5ACon1/i-7mut), and compared the virus propagation and the induction of interferon-stimulated genes (ISGs). By transfecting RNAs of these strains into HuH-7-derived cells, we found that the efficiency of infectious virus production of JFH1/5ACon1/i-7mut was attenuated compared with JFH1/5ACon1. After transfecting full-length HCV RNA into HepaRG cells, the mRNA expression of ISGs was sufficiently induced by IFN treatment in JFH1/5ACon1/i-7mut-transfected but not in JFH1/5ACon1-transfected cells. These data suggested that the NS5A-mediated inhibition of ISG induction was deteriorated by amino acid substitutions in the ISDR. In conclusion, using recombinant JFH-1 viruses, we demonstrated that HCV NS5A is associated with infectious virus production and the inhibition of IFN signaling, and amino acid substitutions in the NS5A ISDR deteriorate these functions. These observations explain the strain-specific evasion of IFN signaling by HCV.

## INTRODUCTION

Hepatitis C virus (HCV) infection is a major cause of chronic liver diseases worldwide. In most cases, HCV establishes a persistent infection and leads to cirrhosis and hepatocellular carcinoma [1, 2]. To eradicate HCV, interferon (IFN)-based treatments have been used for the past two decades; however, the treatment efficacies have not been satisfactory [3, 4]. The inhibition of immune responses such as attenuation of the IFN-signaling cascade by this virus is thought to be responsible for this insufficient efficacy of IFN-based treatments. Recently, the novel anti-HCV therapies with direct-acting antivirals (DAAs) have achieved viral clearance more than 90% of chronic hepatitis C patients [5–7]. However, several treatment-refractory cases have been reported, and, in such cases, the evasion strategies against immune system are concerned. Therefore, it is still important to evaluate the molecular mechanisms that responsible for IFN resistance to understand the strategy the virus uses to establish a persistent infection.

HCV, a member of the *Hepacivirus* genus, is an enveloped virus with a positive single-stranded RNA genome of approximately 9600 nucleotides [8]. This virus has a single large open reading frame that encodes structural proteins (core, E1, and E2) for virus particle components and nonstructural (NS) proteins (p7, NS2, NS3, NS4A, NS4B, NS5A and NS5B) that are associated with virus replication [9]. Among these proteins, NS5A is known to be multi-functional. This protein modulates HCV propagation by forming the replication complex with other NS proteins and certain host factors and is also involved in infectious viral particle production through its interaction with the core protein [10–13]. NS5A has also been implicated in various forms of viral pathogenesis through its interactions with a variety of host cellular proteins. Moreover, it has been suggested that NS5A is linked to the outcome of IFN-based treatment. Historically, Enomoto *et al.* first reported that the virus genome sequences in a certain region (aa 2209 to 2248) in NS5A of HCV genotype 1b strains can predict the outcome of IFN treatment and named this region IFN sensitivity-determining region (ISDR) [14, 15]. They compared the amino acid sequence of this region in patient’s HCV with the reference HCV strain (HCV-J), and found that the number of substitutions were associated with the outcome of IFN treatment. After that, the association between IFN response and amino acid substitutions in the ISDR was studied extensively and was verified by numerous clinical studies, including studies on the use of pegylated-IFN treatments or the addition of Ribavirin [16–20]. However, the underlying mechanisms are still unclear.

In this study, we aimed to assess the involvement of amino acid substitutions in the ISDR on the HCV life cycle and to clarify the molecular mechanisms responsible for the ISDR-associated effects on IFN susceptibility using an HCV cell culture system. Because clinical studies on the ISDR have primarily been performed in patients infected with HCV genotype 1b HCV strains, we used the recombinant JFH-1 virus (genotype 2a) and replaced its NS5A gene with NS5A of the genotype 1b Con1 strain.

## RESULTS

### Amino acid substitutions in the ISDR affect infectious virus production

**Figure 1:**
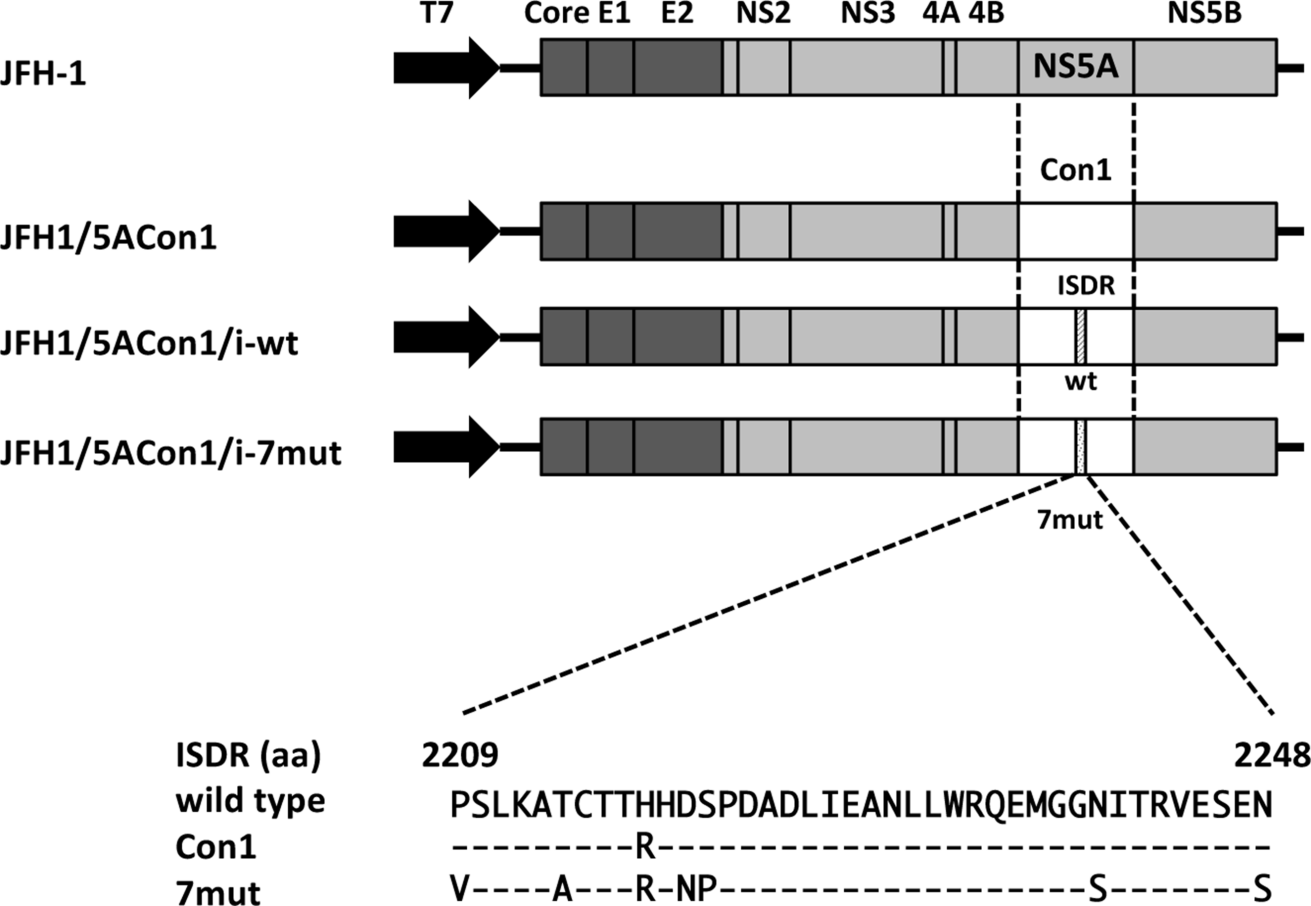
Schematic structures of recombinant JFH-1 viruses and alignment of amino acids in the ISDR Structures of recombinant JFH1 viruses carrying NS5A of a genotype 1b strain were indicated. We generated 3 JFH-1-based recombinant viruses: JFH1/5ACon1 (carrying NS5A of the genotype 1b Con1 strain), JFH1/5ACon1/i-wt (containing wild-type amino acids in the ISDR), and JFH1/5ACon1/i-7mut (containing 7 patient-derived amino acid substitutions in the ISDR). An alignment of amino acids in the ISDRs used in this study was also indicated. Con1 has 1 amino acid substitution and 7mut has 7 amino acid substitutions compared with the wild-type ISDR.

**Figure 2:**
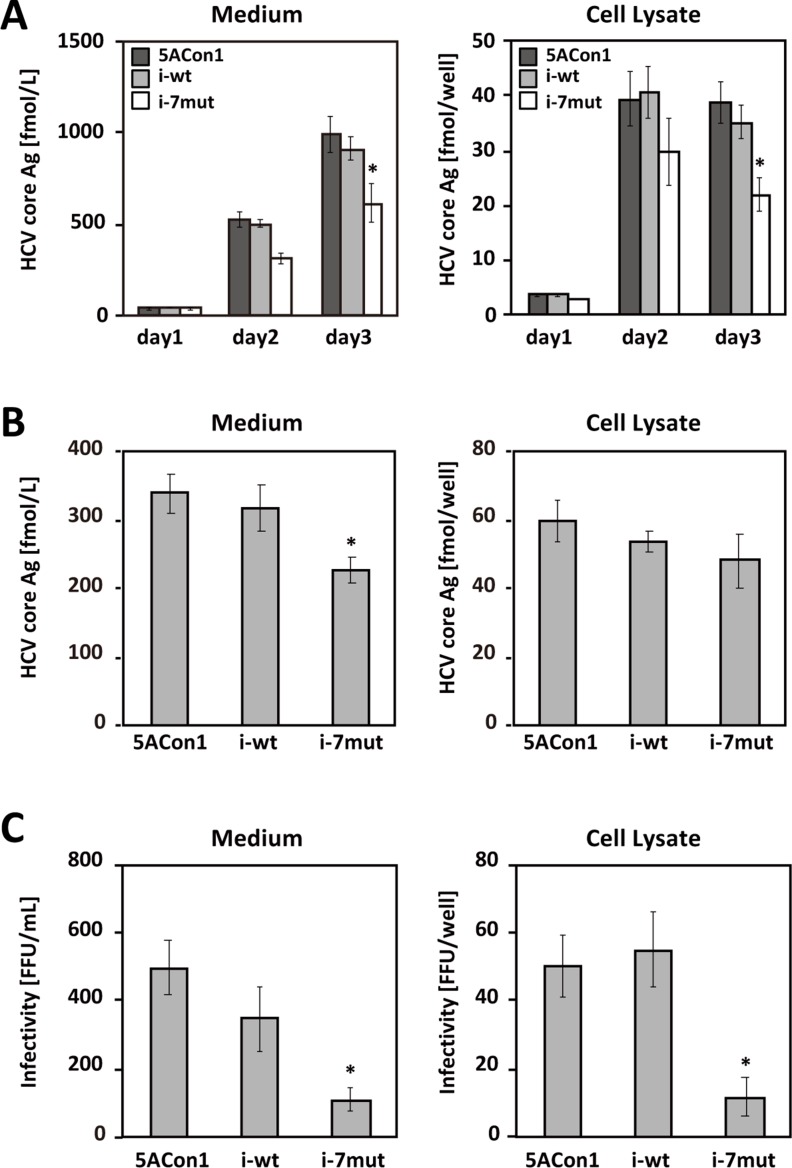
Replication and virus production of recombinant JFH-1 viruses (**A**) Huh-7.5.1 cells were transfected with the full-length RNAs of JFH1/5ACon1 (dark-gray bar), JFH1/5ACon1/i-wt (light-gray bar), and JFH1/5ACon1/i-7mut (white bar). The levels of the HCV core Ag in the medium and cell lysate were determined at the indicated time points ^*^P < 0.05. (**B**) Huh7-25 cells were transfected with the full-length RNAs of JFH1/5ACon1 (5ACon1), JFH1/5ACon1/i-wt (i-wt), and JFH1/5ACon1/i-7mut (i-7mut). Three days after transfection, the levels of the HCV core Ag in the medium and cell lysate were determined ^*^P < 0.05. (**C**) The infectivity titers in the medium and cell lysate were also determined ^*^P < 0.05.

To elucidate the effects of the ISDR on the HCV life cycle, we created 3 recombinant JFH-1 viruses: JFH1/5ACon1 (containing NS5A of the Con1 strain with 1 amino acid difference in the ISDR compared with that of the HCV-J strain), JFH1/5ACon1/i-wt (containing the ISDR of the HCV-J strain), and JFH1/5ACon1/i-7mut (containing 7 amino acid substitutions in the ISDR compared with that of the HCV-J strain) (Figure 1). These introduced mutations came from the ISDR sequence of the HCV strain in an acute hepatitis C patient [21]. To assess the propagation of these viruses, we transfected the full-length RNAs of these strains into Huh-7.5.1 cells and measured the intra- and extra-cellular HCV core antigen (Ag) levels at days 1, 2, and 3 post-transfection. Both the intra- and extra-cellular HCV core Ag levels in these cells gradually increased in a time-dependent manner, indicating that these recombinant viruses were capable of replicating in Huh-7.5.1 cells. At day 3 post-transfection, the core Ag levels of JFH1/5ACon1/i-7mut were significantly lower than those of JFH1/5ACon1 and JFH1/5ACon1/i-wt in the culture medium and in cell lysates (Figure 2A).

To assess the effect of the ISDR amino acid substitutions on each step of the HCV life cycle, we performed single-cycle virus production assays using the CD81-negative cell line Huh7–25 [22]. This cell line supports only replication and infectious virus production upon transfection of full-length HCV RNA, but it cannot be re-infected by produced HCV. Thus, it allows the assessment of a single cycle of infectious viral production [23–25]. After transfection of full-length HCV RNA, the replication level of HCV can be assessable by measuring the intracellular HCV core Ag levels, and the efficiency of infectious virus production can be estimable by evaluating the intracellular infectivity titers. Three days after the full-length RNAs of these strains were transfected into Huh7–25 cells, the intra- and extra-cellular HCV core Ag levels and infectivity titers were measured. Similar to the data obtained using Huh-7.5.1 cells, the core Ag level of JFH1/5ACon1/i-7mut was significantly lower than that of JFH1/5ACon1 or JFH1/5ACon1/i-wt in the culture medium; however, the levels in the cell lysates were comparable (Figure 2B). The intra- and extra-cellular infectivity titers of JFH1/5ACon1/i-7mut were also significantly lower than those of JFH1/5ACon1 and JFH1/5ACon1/i-wt (Figure 2C). These data suggested that the replication level of the viruses was comparable but that the production of infectious JFH1/5ACon1/i-7mut virus was attenuated compared with the JFH1/5ACon1 and JFH1/5ACon1/i-wt viruses, and this lower virus production was associated with a lower core Ag level in the culture medium of JFH1/5ACon1/i-7mut-transfected cells.

### Amino acid substitutions in the ISDR reduce the interaction and co-localization of NS5A and core

**Figure 3:**
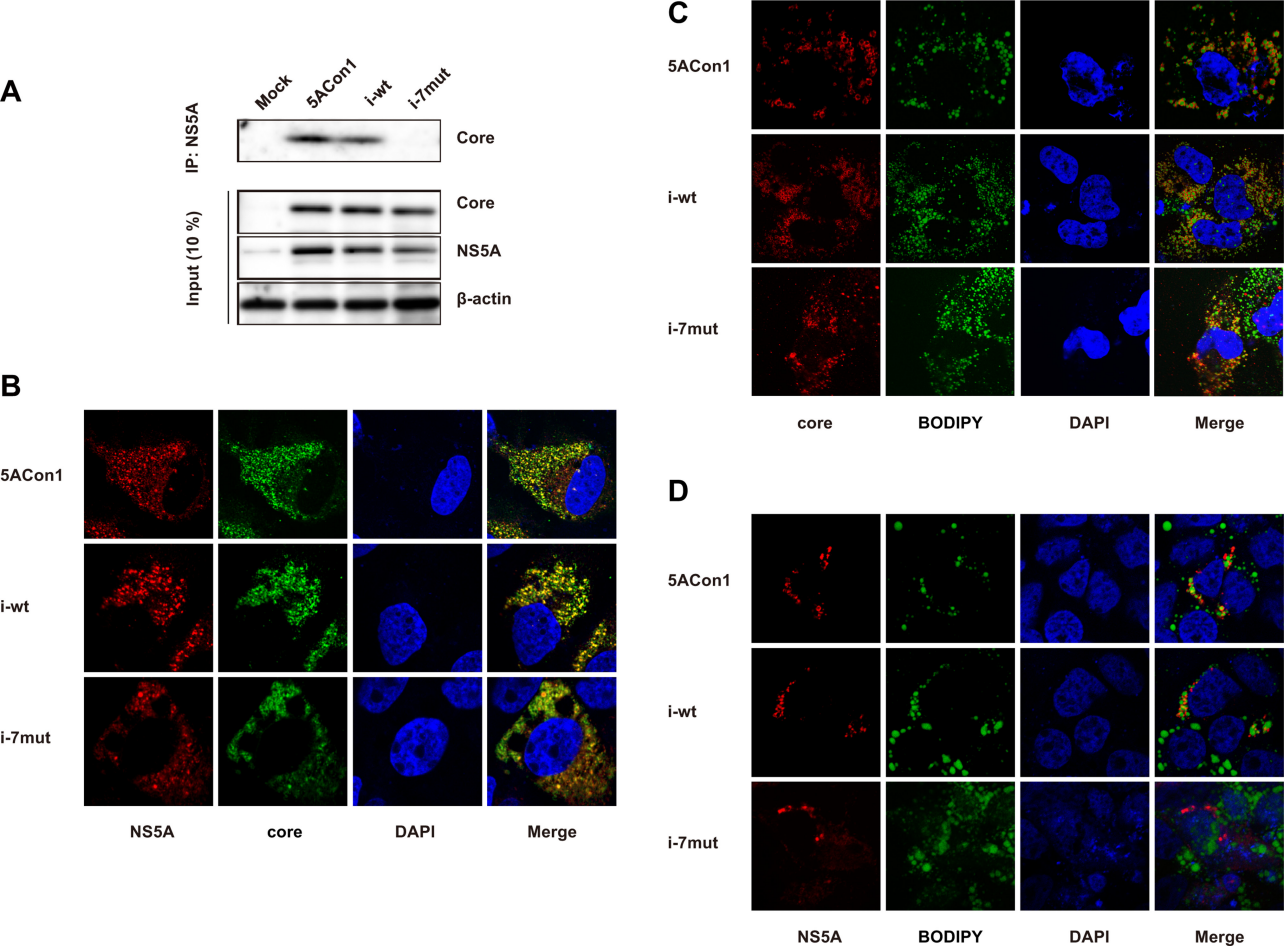
Effect of amino acid substitutions in the ISDR on the interaction between the NS5A and core proteins (**A**) Huh-7.5.1 cells were transfected with the full-length RNAs of JFH1/5ACon1, JFH1/5ACon1/i-wt, and JFH1/5ACon1/i-7mut. At 48 h post-transfection, the cell lysates were immunoprecipitated with an anti-NS5A antibody, followed by immunoblotting analysis with an anti-core antibody. (**B**) Huh-7.5.1 cells were transfected with the full-length RNAs of JFH1/5ACon1 (5ACon1), JFH1/5ACon1/i-wt (i-wt), and JFH1/5ACon1/i-7mut (i-7mut). At 48 h post-transfection, the cells were stained with anti-NS5A and anti-core antibodies and analyzed by fluorescence microscopy. Red: NS5A; Green: core; Blue: DAPI. (**C**) Huh-7.5.1 cells were transfected with the indicated full-length RNAs. At 48 h post-transfection, the cells were stained with an anti-core antibody and BODIPY and analyzed by fluorescence microscopy. Red: core; Green: Lipid droplets; Blue: DAPI. (**D**) Huh-7.5.1 cells were transfected with the indicated full-length RNAs. At 48 h post-transfection, the cells were stained with an anti-NS5A antibody and BODIPY and analyzed by fluorescence microscopy. Red: NS5A; Green: Lipid droplets; Blue: DAPI.

To investigate the molecular mechanisms responsible for the attenuated production of infectious JFH1/5ACon1/i-7mut virus, we assessed the interaction between the HCV core and NS5A proteins. We transfected the full-length RNAs of JFH1/5ACon1, JFH1/5ACon1/i-wt and JFH1/5ACon1/i-7mut into Huh-7.5.1 cells, and the amount of NS5A-associated core protein was detected after immunoprecipitation with an anti-NS5A antibody. At 48 h post-transfection, the co-immunoprecipitated core protein was detected in JFH1/5ACon1- and JFH1/5ACon1/i-wt-transfected cells. However, the core protein was not co-immunoprecipitated in JFH1/5ACon1/i-7mut-transfected cells (Figure 3A). To assess the co-localization of the core and NS5A proteins, we transfected cells with the full-length RNAs of these strains and then analyzed protein localization by immunostaining. Upon staining with anti-core and anti-NS5A antibodies, co-localization of these proteins was detected in JFH1/5ACon1- and JFH1/5ACon1/i-wt-transfected cells. However, this co-localization was not observed in JFH1/5ACon1/i-7mut-transfected cells (Figure 3B). To assess the co-localization of core and lipid droplets (LDs) or NS5A and LDs, we also co-stained the cells with an anti-core or anti-NS5A antibody and BODIPY. Upon staining with an anti-core antibody and BODIPY, the core protein was localized to the areas surrounding LDs in all transfected cells (Figure 3C). Staining with an anti-NS5A antibody and BODIPY also revealed that the NS5A protein could be detected in the areas surrounding LDs in JFH1/5ACon1- and JFH1/5ACon1/i-wt-transfected cells. However, in JFH1/5ACon1/i-7mut-transfected cells, the NS5A protein did not localize near LDs (Figure 3D).

### Effects of amino acid substitutions in the ISDR on IFN susceptibility in Huh-7.5.1 cells

**Figure 4:**
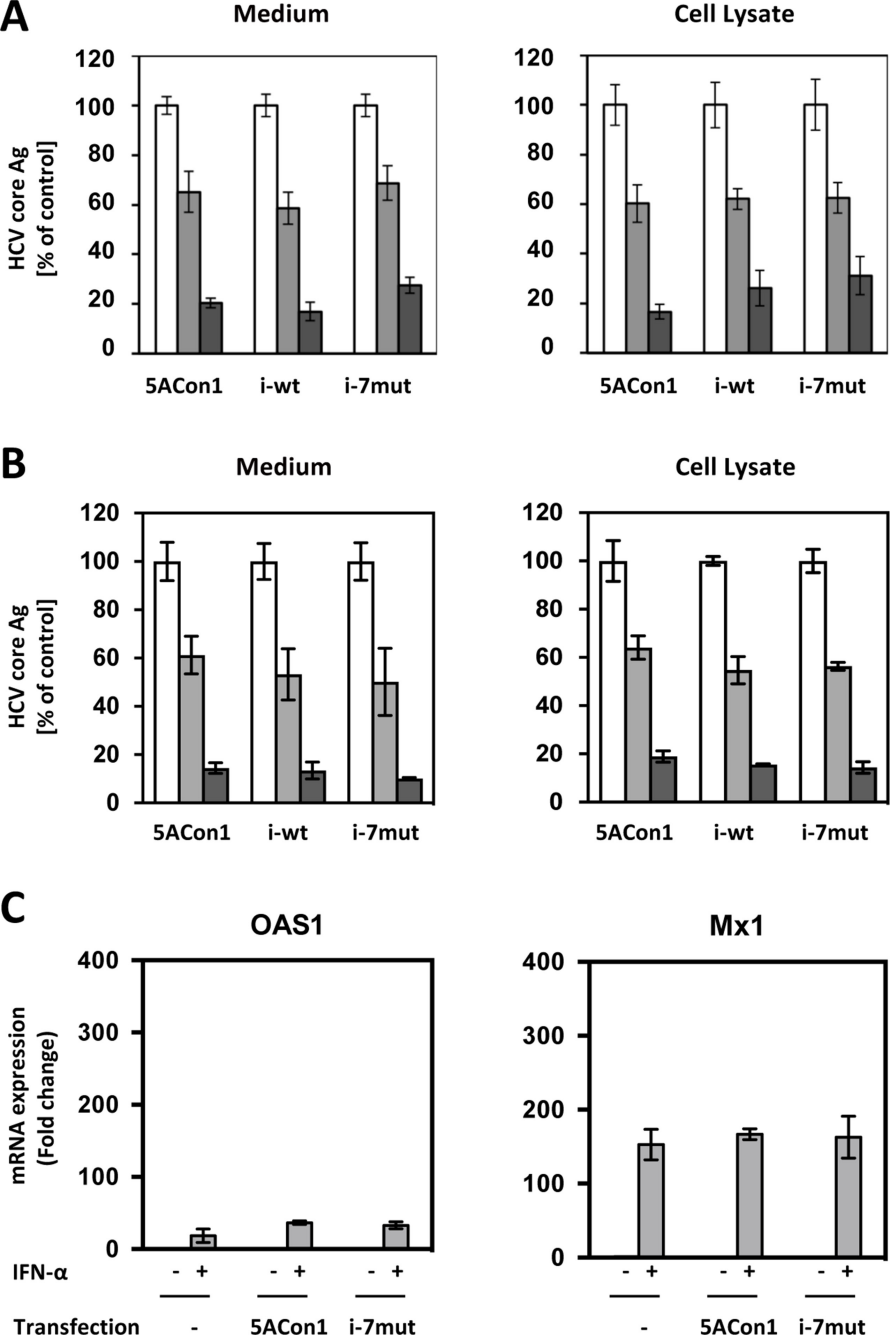
Effect of amino acid substitutions in the ISDR on IFN-α susceptibility in Huh-7.5.1 cells (**A**) Huh-7.5.1 cells were transfected with the indicated full-length RNAs of JFH1/5ACon1 (5ACon1), JFH1/5ACon1/i-wt (i-wt), and JFH1/5ACon1/i-7mut (i-7mut). At 4 h post-transfection, the cells were treated with 10 IU/mL (light-gray bar) or 100 IU/mL (dark-gray bar) IFN-α or diluent (white bar). At 48 h post-transfection, the levels of HCV Core Ag in the medium and cell lysate were measured. (**B**) Huh-7.5.1 cells were transfected with the indicated full-length RNAs of JFH1/5ACon1 (5ACon1), JFH1/5ACon1/i-wt (i-wt), and JFH1/5ACon1/i-7mut (i-7mut). At 4 h post-transfection, the cells were treated with 3 pM (light-gray bar) or 10 pM (dark-gray bar) Daclatasvir or diluent (white bar). At 48 h post-transfection, the levels of HCV Core Ag in the medium and cell lysate were measured. (**C**) Huh-7.5.1 cells were transfected with the full-length RNAs of JFH1/5ACon1 and JFH1/5ACon1/i-7mut. At 4 h post-transfection, the cells were treated with IFN-α or diluent. After 20 h, the cells were harvested, and the mRNA expression levels of the indicated ISGs were measured.

To investigate the effects of amino acid substitutions in the ISDR on IFN susceptibility, Huh-7.5.1 cells were transfected with the full-length RNAs of JFH1/5ACon1, JFH1/5ACon1/i-wt and JFH1/5ACon1/i-7mut and then treated with IFN-α, and the intra- and extra-cellular HCV core Ag levels were measured. After 48 h of treatment with IFN-α at the concentrations of 10 and 100 IU/mL, the intra- and extra-cellular HCV core Ag levels were reduced in a dose-dependent manner (Figure 4A). However, the reductions in the HCV core Ag levels were comparable among the strains. The effect of amino acid substitutions in the ISDR on the susceptibility to the NS5A inhibitor, Daclatasvir, was also evaluated, and similar reductions of HCV core Ag were observed (Figure 4B). To elucidate the effects of amino acid substitutions in the ISDR on interferon-stimulated genes (ISGs) inductions, we transfected the full-length RNAs of recombinant JFH1 viruses into Huh-7.5.1 cells and measured the mRNA induction of 2’,5’-oligoadenylate synthetase 1 (OAS1) and myxovirus resistance gene 1 (Mx1). In this experiment, we compared JFH1/5ACon1/i-7mut with JFH1/5ACon1, because JFH1/5ACon1 and JFH1/5ACon1/i-wt exhibited similar characteristics in the earlier studies of HCV propagation, infectious virus production and subcellular localization of the NS5A protein. As expected, the mRNA inductions of OAS1 and Mx1 were not detected by transfection of full-length RNAs of recombinant JFH1 viruses because the critical mutation in retinoic acid-inducible gene-I (RIG-I) gene has been reported. After IFN-α treatment, we could detect the mRNA inductions of OAS1 and Mx1, but the induction levels were not different between JFH1/5ACon1- and JFH1/5ACon1/i-7mut-transfected cells (Figure 4C). Therefore, we concluded that Huh-7.5.1 cells is not suitable to investigate the effect of amino acid substitutions in the ISDR on IFN susceptibility.

### Effects of amino acid substitutions in the ISDR on IFN susceptibility in HepaRG cells

**Figure 5:**
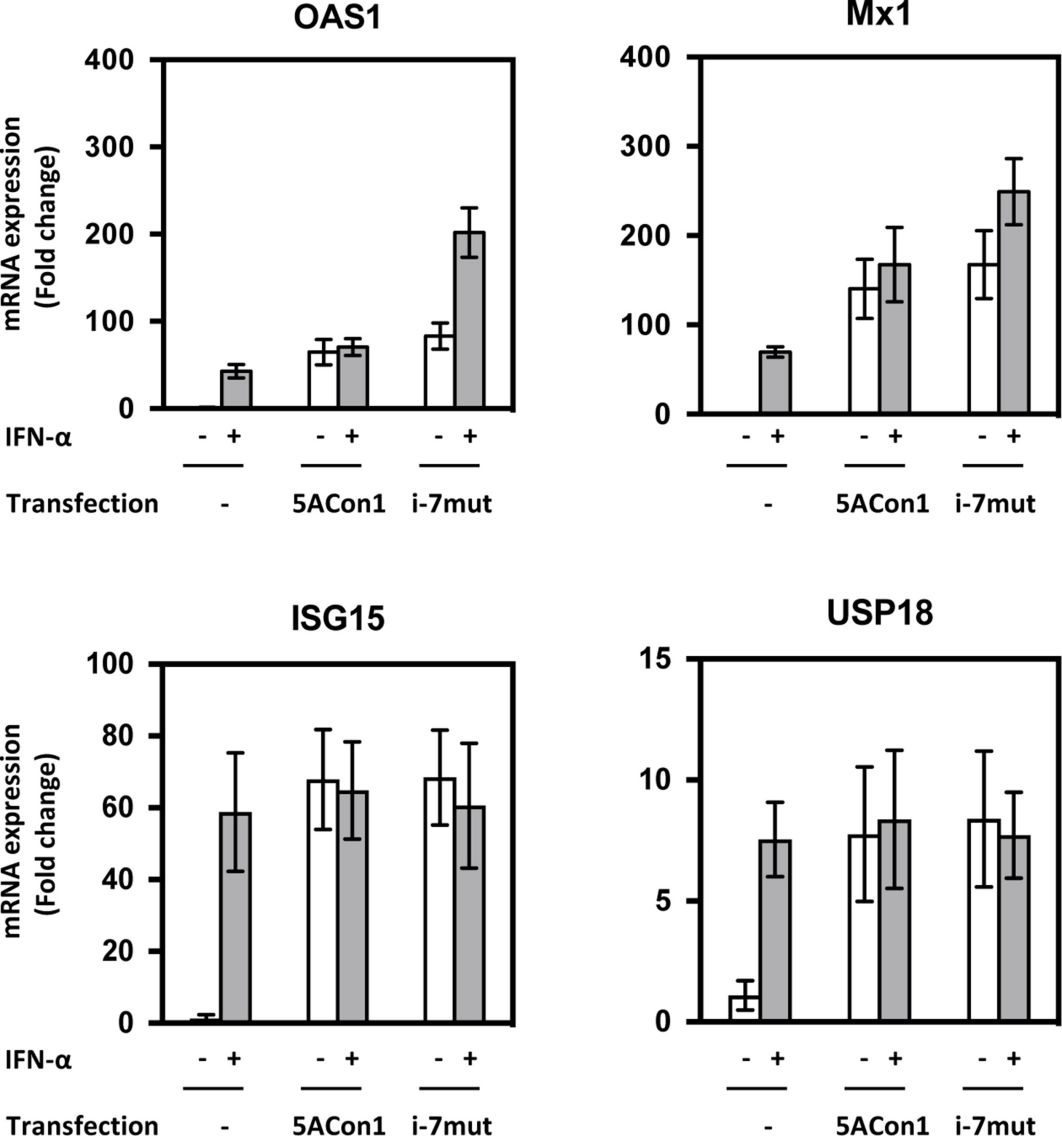
Effects of amino acid substitutions in the ISDR on ISG induction in HepaRG cells HepaRG cells were transfected with the full-length RNAs of JFH1/5ACon1 and JFH1/5ACon1/i-7mut. At 4 h post-transfection, the cells were treated with IFN-α or diluent. After 20 h, the cells were harvested, and the mRNA expression levels of the indicated ISGs were measured.

To assess the precise effects of amino acid substitutions in the ISDR on IFN susceptibility, we exploited HepaRG cells, which are physiologically similar to differentiated hepatocytes and maintain the characteristics of hepatocytes. We transfected the full-length RNAs of recombinant JFH1 viruses into HepaRG cells and measured the mRNA induction of OAS1 and Mx1. After transfection with the full-length RNAs of JFH1/5ACon1 and JFH1/5ACon1/i-7mut, the mRNA expression of OAS1 and Mx1 was induced at comparable levels (Figure 5). When JFH1/5ACon1/i-7mut-transfected cells were treated with 10 IU/mL IFN-α, the expression levels of these ISGs were enhanced. However, this enhanced expression was attenuated in JFH1/5ACon1-transfected cells. Thus, in this cell line, the difference for inductions of OAS1 and Mx1 by IFN treatment was clearly indicated between JFH1/5ACon1- and JFH1/5ACon1/i-7mut-transfected cells. The inductions of un-phosphorylated IFN-stimulated gene factor 3 (U-ISGF3) -associated ISGs, IFN-stimulated gene 15 (ISG15) and ubiquitin-specific protease 18 (USP18), were also evaluated. The expression levels of these ISGs were similar in JFH1/5ACon1- and JFH1/5ACon1/i-7mut-transfected cells, and not affected by amino acid substitutions in the ISDR.

**Figure 6:**
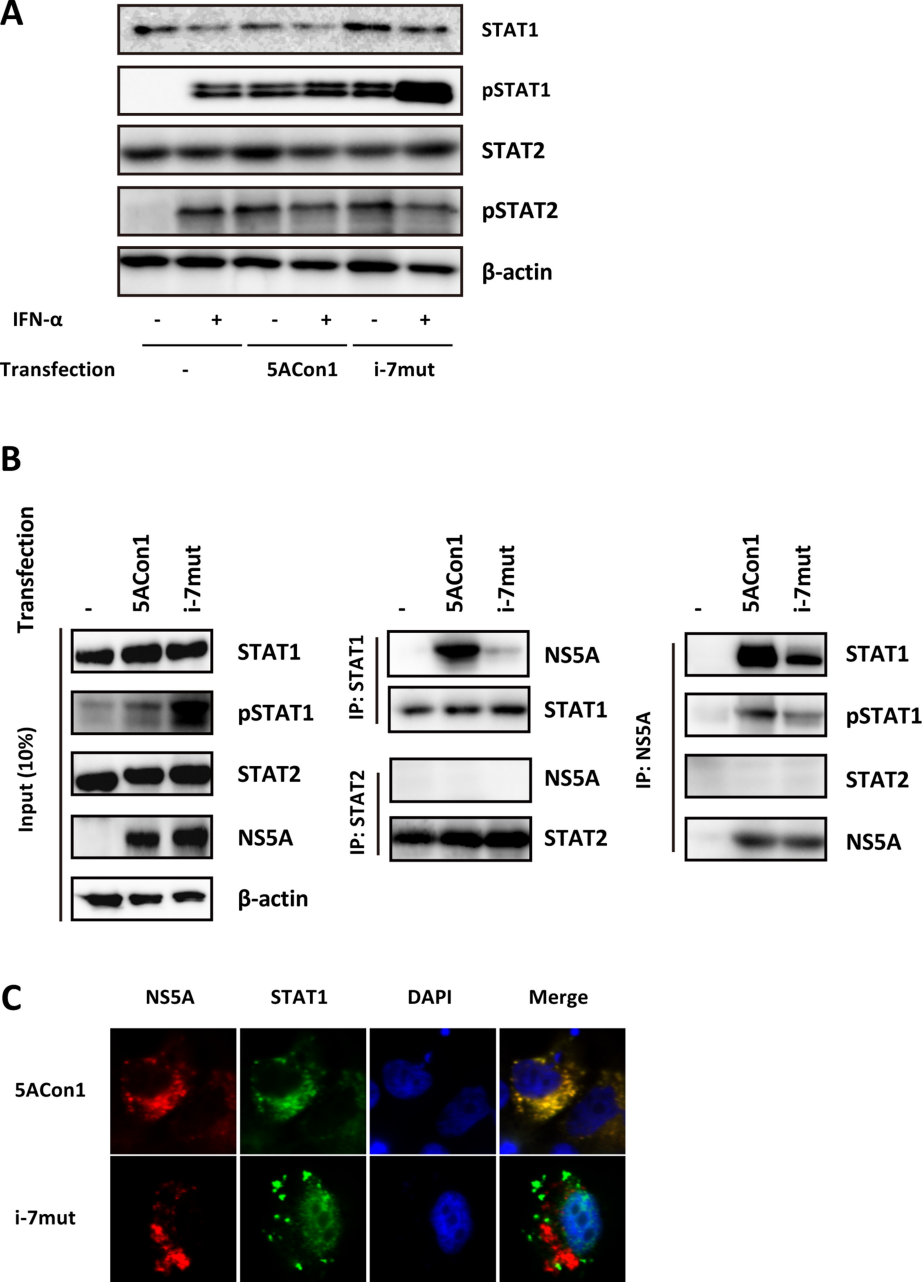
Effects of amino acid substitutions in the ISDR on STAT1 phosphorylation (**A**) HepaRG cells were transfected with the full-length RNAs of JFH1/5ACon1 and JFH1/5ACon1/i-7mut. At 48 h post-transfection, the cells were treated with IFN-α or diluent. After 30 min, the cell lysates were subjected to immunoblotting analysis with the indicated antibody. (**B**) HepaRG cells were transfected with NS5A expression vectors (5ACon1 or 5ACon1/i-7mut) or an empty vector. At 48 h post-transfection, the cells were treated with IFN-α. After 30 min, the cell lysates were immunoprecipitated with anti-STAT1, anti-STAT2, or anti-NS5A antibodies, followed by immunoblotting analysis with the indicated antibodies. (**C**) HepaRG cells were transfected with NS5A expression vectors (5ACon1 or 5ACon1/i-7mut). At 48 h post-transfection, the cells were treated with IFN-α. After 30 min, the cells were stained with the indicated antibodies and analyzed by fluorescence microscopy. Red: NS5A; Green: STAT1; Blue: DAPI.

To clarify the molecular mechanisms responsible for the different effects on ISGs mediated by these strains, we evaluated the phosphorylation of STAT1. Upon treatment with 100 IU/mL IFN-α, an increased amount of phosphorylated STAT1 (pSTAT1) was detected in JFH1/5ACon1/i-7mut-transfected cells, whereas this increase was not observed in JFH1/5ACon1-transfected cells (Figure 6A). The amount of phosphorylated STAT2 (pSTAT2) were comparable in these cells. To examine whether NS5A and STAT1 directly interact, NS5A expression vectors (pCAG/5ACon1 or pCAG/5ACon1/i-7mut) were transfected into HepaRG cells, and the interaction between NS5A and STAT1 was assessed by immunoprecipitation. After immunoprecipitation with an anti-STAT1 antibody, a large amount of STAT1-associated NS5A protein was detected in pCAG/5ACon1-transfected HepaRG cells (Figure 6B). However, very little STAT1-associated NS5A was detected in pCAG/5ACon1/i-7mut-transfected cells, suggesting that NS5A carrying an ISDR with amino acid substitutions has a lower affinity for STAT1 than NS5A carrying the Con1 ISDR. We also evaluated the interaction between NS5A and STAT2. However, this interaction was not observed in both pCAG-5ACon1- and pCAG-5ACon1/i-7mut- transfected cells (Figure 6B). These observations were confirmed by immunoprecipitation with an anti-NS5A antibody and detection with anti-STAT1, anti-pSTAT1, and anti-STAT2 antibodies. The differences in the interaction of these proteins and the translocation of pSTAT1 were also elucidated by immunostaining. Co-localization of NS5A and STAT1 was detected in the cytoplasm of pCAG/5ACon1-transfected HepaRG cells. However, the STAT1 protein in pCAG/5ACon1/i-7mut-transfected cells did not co-localize with the NS5A protein and instead translocated into the nucleus (Figure 6C).

## DISCUSSION

In 1986, the treatment of chronic hepatitis C patients (designated non-A non-B hepatitis at that time) with IFN-α was first reported [26]. Because the efficacy of IFN-α treatment was insufficient, studies were conducted to identify improvements in reagents and treatment regimens. Although the administration of IFN with Ribavirin or the use of pegylated-IFN improved the rate of sustained virologic response, virus eradication was not achieved in all HCV-infected patients. Therefore, substantial effort was directed toward identifying patients who were susceptible to IFN-based treatment, and several host factors, such as SNPs in IFN-λ3 and/or IFN-λ4 locus, or viral factors, such as amino acid polymorphisms in core region or amino acid substitutions in the ISDR, were identified as predictors for the outcome of IFN-based treatment. Among these, the discovery of ISDR was the first and its usefulness was verified in several clinical studies [15–19]. However, the underlying mechanisms have not yet been determined. ISDR-related studies are difficult for two reasons. First, the IFN-based treatment outcome predictions were primarily performed in patients infected with HCV genotype 1b strains. However, the replication-competent HCV strain JFH-1 is genotype 2a, and an infection and replication system using genotype 1b strains is currently unavailable. Thus, it is very difficult to evaluate the effects of the ISDR of genotype 1b strains on the HCV life cycle or IFN susceptibility in cell culture. We overcame this difficulty by using a recombinant JFH-1 virus containing the NS5A gene of a genotype 1b strain (JFH1/5ACon1). Second, efficient HCV replication is only observed in HuH-7 and its derivative cell lines, and HCV replication or infectious virus production in primary human hepatocytes is limited. The HuH-7 cell line and its derivatives exhibit some considerable defects in the innate immune system, and these defects might hamper the assessment of IFN susceptibility. In this study, to assess the effect of amino acid substitutions in the ISDR on IFN susceptibility, we exploited HepaRG cells, which are known to be physiologically similar to hepatocytes and to maintain the characteristics of hepatocytes [27].

By analyzing the recombinant JFH-1 viruses containing NS5A of a genotype 1b strain, we demonstrated the attenuated production of infectious JFH1/5ACon1/i-7mut virus but did not observe effects on its replication compared with the JFH1/5ACon1 and JFH1/5ACon1/i-wt viruses. Although replication efficiency has been reportedly enhanced by the introduction of amino acid substitutions in the ISDR using the subgenomic replicon system with a genotype 1b strain, this enhancement was not observed in our system using full-length recombinant JFH-1 viruses [28]. This discrepancy may be due to the different systems used. The addition of the structural region and the requirement of virus particle assembly may mask any enhanced replication. Impaired infectivity and propagation abilities by induction of amino acid substitutions in the ISDR were also reported in an *in vivo* HCV infection model using human hepatocyte-transplanted uPA-SCID mice [29]. To produce infectious viruses, the interaction of the HCV core and NS5A proteins and their co-localization with LDs have been reported to be essential [13, 30–32]. Using immunoprecipitation, we demonstrated that the interaction between the core and NS5A proteins is abolished in JFH1/5ACon1/i-7mut-transfected cells, while this interaction was clearly detected in JFH1/5ACon1- and JFH1/5ACon1/i-wt-transfected cells. The subcellular localizations of these proteins were also affected by amino acid substitutions in the ISDR. As reported by Miyanari *et al.*, the core proteins localize around LDs, and NS5A localizes to the areas surrounding the core proteins and LDs [30]. We could detect the co-localization of the core and NS5A proteins and the localization of these proteins around LDs in JFH1/5ACon1- and JFH1/5ACon1/i-wt-transfected cells. However, the co-localization of the core and NS5A proteins was disrupted and the NS5A protein did not localize near LDs in JFH1/5ACon1/i-7mut-transfected cells. These observations suggested that the weak interaction between core and NS5A carrying an ISDR with amino acid substitutions and the altered subcellular localization of these proteins reduced the efficiency of infectious virus production, and, as a result, JFH1/5ACon1/i-7mut has a lower propagation ability compared with JFH1/5ACon1 and JFH1/5ACon1/i-wt. A lower population of viruses carrying the mutant-type ISDR has been reported [15]. Genotype 1b strains with a mutant-type ISDR (more than 4 amino acid substitutions) were detected in 16/84 (19.0%) patients, whereas genotype 1b strains with the wild-type ISDR (no substitution) or an intermediate-type ISDR (1 – 3 substitutions) were detected 30/84 (35.7%) and 38/84 (45.2%) patients, respectively. The low propagation efficiency of the JFH1/5ACon1/i-7mut virus may explain this difference in populations.

We expected that these differences in the subcellular localization of the NS5A protein and the interaction between the NS5A and core proteins might be associated with IFN susceptibility. Thus, we compared the susceptibilities to IFN among the recombinant JFH-1 viruses. However, we could not detect any differences in virus replication inhibition among these viruses. We hypothesized that this discrepancy with the clinical observations might be due to the characteristics of Huh-7.5.1 cells. This cell line is known to be highly permissive for HCV replication, and it exhibits some considerable defects in the innate immune system. Therefore, we exploited HepaRG cells, which are known to be physiologically similar to differentiated hepatocytes [27]. To assess the IFN susceptibilities of these recombinant JFH-1 viruses, we evaluated the replication of these strains after the transfection of full-length RNAs into HepaRG cells. However, we could not detect sufficient replication of these viruses in this cell line even without IFN administration (data not shown). A similar observation has been reported previously [33].

IFN is known to elicit its anti-viral effects by activating the IFN signaling cascade through cellular receptors. STAT1 is a key molecule in the IFN signaling pathway. This molecule is phosphorylated and translocated into the nucleus after binding with STAT2 and IFN-regulatory factor 9 (IRF9), leading to the induction of ISGs. Thus, the level of pSTAT1 is considered to represent the effect of IFN. In addition, it has been reported that the HCV NS5A protein plays a role in the inhibition of IFN signaling [34–38]. Thus, we evaluated the effects of amino acid substitutions in the ISDR on the induction of ISGs by IFN-α. In HepaRG cells, the induction of ISGs was observed after transfection of the full-length RNAs of recombinant JFH-1 viruses. Although this induction was substantially enhanced by IFN-α treatment in JFH1/5ACon1/i-7mut-transfected cells, this enhancement was totally absent in JFH1/5ACon1-transfected cells. This observation suggests that the NS5A protein carrying the Con1 ISDR can inhibit the IFN-mediated induction of ISGs but that this inhibition was blocked by amino acid substitutions in the ISDR and therefore not observed in JFH1/5ACon1/i-7mut-transfected cells. Interestingly, this observation was not reproducible in Huh-7.5.1 cells. The induction of ISGs by HCV RNA transfection was not detectable in this cell line because of mutational inactivation of the gene encoding RIG-I, as reported previously [39]. In addition to this dysfunction, the difference in IFN-mediated ISG induction between JFH1/5ACon1/i-7mut- and JFH1/5ACon1-transfected cells was also undetectable in Huh-7.5.1 cells. Thus, we concluded that HepaRG cells, but not Huh-7.5.1 cells, were suitable for investigations of IFN signaling. To elucidate the molecular mechanisms responsible for the difference in IFN-mediated ISG induction between JFH1/5ACon1/i-7mut- and JFH1/5ACon1-transfected cells, the phosphorylation of STAT1 was evaluated. STAT1 phosphorylation was induced by HCV RNA transfection and enhanced by IFN-α treatment in JFH1/5ACon1/i-7mut-transfected cells. However, this enhancement by IFN-α was inhibited in JFH1/5ACon1-transfected cells. Because the NS5A protein has been reported to inhibit the phosphorylation of STAT1 via a direct interaction, we subsequently evaluated the clone-dependent interaction between the NS5A and STAT1 proteins [38]. The NS5A protein carrying the Con1 ISDR could interact with STAT1 and sequester it in the cytoplasm. However, the NS5A protein carrying an ISDR with amino acid substitutions failed to interact with and anchor STAT1 in the cytoplasm, and the translocation of STAT1 was detected. These data suggested that the NS5A protein can inhibit IFN signaling by directly interacting with STAT1 and anchoring it in the cytoplasm. Amino acid substitutions in the NS5A ISDR block this function and make the virus to susceptible to IFN.

In conclusion, using recombinant JFH-1 viruses, we demonstrated that HCV NS5A is associated with infectious virus production and the inhibition of IFN signaling, and amino acid substitutions in the NS5A ISDR block these functions. These observations may explain the strain-specificity of HCV evasion of IFN signaling and the strain-specific susceptibility to IFN-based treatment.

## MATERIALS AND METHODS

### Cell culture

The HuH-7-derived cell lines Huh-7.5.1, which allows efficient HCV replication (a kind gift from Francis V. Chisari, Scripps Research Institute, La Jolla, CA, USA) [40], and Huh7-25, which lacks CD81 expression as reported previously [22], were cultured at 37°C in a 5% CO_2_ environment in Dulbecco’s Modified Eagle’s Medium containing 10% fetal bovine serum as described previously. HepaRG cells, which are known to be physiologically similar to differentiated hepatocytes, were obtained from Life Technologies (Carlsbad, CA, USA) and cultured according to the manufacturer’s instructions.

### Plasmid construction and RNA synthesis

The plasmid containing the recombinant JFH-1 virus carrying the NS5A gene of the genotype 1b Con1 strain, JFH1/5ACon1, has been described previously [41, 42]. The HCV Con1 strain contains 1 amino acid substitution in the NS5A ISDR compared with the HCV-J strain (the reference strain of the wild-type ISDR; the accession number is D90208). The fragment containing the ISDR sequence of the HCV-J strain was generated by site-directed mutagenesis. The generated fragment was replaced with JFH1/5ACon1 and named JFH1/5ACon1/i-wt (Figure 1). To assess the effects of the introduction of amino acid substitutions in the ISDR, we exploited the ISDR sequence found in an acute hepatitis C patient [21]. This patient-derived ISDR has 7 amino acid substitutions compared with the HCV-J strain. The generated ISDR fragment with these 7 amino acid substitutions was also used to replace the JFH1/5ACon1 ISDR and named JFH1/5ACon1/i-7mut (Figure 1). The full-length HCV RNAs of these strains were synthesized as described previously [43].

To construct the NS5A expression vectors, the fragments of NS5A from JFH1/5ACon1 and JFH1/5ACon1/i-7mut were amplified by PCR and introduced into the pCAG-Neo plasmid (WAKO, Osaka, Japan). The generated NS5A expression vectors were named pCAG/5ACon1 and pCAG/5ACon1/i-7mut.

### RNA transfection and quantification of the HCV core antigen

*In vitro*-transcribed full-length HCV RNAs of these plasmids were electroporated into Huh-7.5.1 or Huh7-25 cells [43]. Seventy-two hours after transfection, the supernatants and cells were harvested and used to measure the HCV core Ag. The concentration of the HCV core Ag was determined by the Lumipulse Ortho HCV Ag kit (Ortho Clinical Diagnostics, Tokyo, Japan) using LUMIPULSE G1200, a fully automated chemiluminescent enzyme immunoassay system (Fujirebio, Tokyo, Japan), according to the manufacturer’s instructions [44]. To assess the susceptibility to IFN-α, IFN-α2b (Intron A, MSD K.K., Tokyo, Japan) was used. To assess the susceptibility to the NS5A inhibitor, Daclatasvir was obtained from ChemScene (Monmouth Junction, NJ, USA).

### Titration of HCV infectivity

The infectivity of the generated HCVs was evaluated by indirect immunostaining as described previously [43]. The infectivity titer was expressed as focus-forming units (FFU) per mL. The intra-cellular infectivity titers were also determined as described previously [43].

### Immunoprecipitation

Transfected cells were resuspended in lysis buffer (50 mM Tris-HCl, pH 7.5, 150 mM NaCl, 1% Nonidet P-40, and 10% glycerol) and incubated with an anti-NS5A antibody and 30 μL of Dynabeads Protein G (Life Technologies). The beads were washed with PBS containing 0.02% Tween 20. The immunocomplexes were eluted by boiling with 20 μL of 2 × sample buffer and analyzed by immunoblotting.

### Immunoblotting

Cells were lysed in lysis buffer (20 mM Tris-HCl, pH 7.5, 200 mM NaCl, and 1% TritonX-100). The proteins were separated by 10% sodium dodecyl sulfate-polyacrylamide gel electrophoresis (SDS-PAGE) and transferred to polyvinylidene fluoride membranes (Merck Millipore, Billerica, MA, USA). The immunoblots were probed with an anti-core antibody (clone 2H9) [45], anti-NS5A antibody (clone KS0265-1) [46], anti-STAT1 antibody (Santa Cruz Biotechnology, Dallas, TX, USA), anti-pSTAT1 antibody (Santa Cruz Biotechnology), anti-STAT2 antibody (Santa Cruz Biotechnology), anti-pSTAT2 antibody (Cell Signaling Technology, Danvers, MA, USA), and anti-β-actin antibody (Sigma-Aldrich, St. Louis, MO, USA). Horseradish peroxidase (HRP)-labeled secondary antibodies (GE Healthcare, Amersham, UK), EzWestLumi Plus HRP detection reagents (ATTO, Tokyo, Japan) and an ImageQuant LAS 4000 Mini lumino image analyzer (GE Healthcare) were used to detect the signals.

### Immunofluorescence and fluorescence microscopy

Cells grown on cover slips were transfected with HCV RNA. The transfected cells were labeled with primary antibodies and Alexa-conjugated secondary antibodies and analyzed via confocal laser microscopy (Olympus, Tokyo, Japan). To detect the localization of LDs, BODIPY was used.

### RNA transfection and gene expression assay

*In vitro*-transcribed full-length HCV RNAs were transfected into Huh-7.5.1 or HepaRG cells using Lipofectamine MessengerMAX Reagent (Life Technologies) following the manufacturer’s instructions. The transfected cells were harvested, and the total cellular RNA was isolated using an RNeasy Mini RNA isolation kit (QIAGEN, Hilden, Germany) according to the manufacturer’s instructions. The total cellular RNA was reverse transcribed using the SuperScript VILO cDNA synthesis kit (Life Technologies) following manufacturer’s instructions. The mRNA expression levels of representative ISGs, OAS1, Mx1, ISG15, and USP18, were quantified using TaqMan Gene Expression Assays (Life Technologies). The expression levels of these ISGs were calculated relative to the mRNA expression level of the internal control, 18S ribosomal RNA (18S rRNA), in the same sample.

### Statistical analysis

Key experiments were performed at least three times. The results are shown as the means ± SD, and the statistical analysis was performed using a paired Student’s *t* test. A *p*-value of < 0.05 was considered significant. At least three replicates were performed for each experiment.

## Abbreviations

HCV: hepatitis C virus
IFN: interferon
ISDR: IFN sensitivity-determining region
ISGs: interferon-stimulated genes
DAAs: direct-acting antivirals
NS: nonstructural
Ag: antigen
LDs: lipid droplets
OAS1: 2’,5’-oligoadenylate synthetase 1
Mx1: myxovirus resistance gene 1
IRF9: IFN-regulatory factor 9
U-ISGF3: un-phosphorylated IFN-stimulated gene factor 3
ISG15: IFN-stimulated gene 15
USP18: ubiquitin-specific protease 18
pSTAT1: phosphorylated STAT1
RIG-I: retinoic acid-inducible gene-I
FFU: focus-forming units
SDS-PAGE: sodium dodecyl sulfate-polyacrylamide gel electrophoresis
18S rRNA: 18S ribosomal RNA
